# Experiences and support needs of consultant psychiatrists following a patient-perpetrated homicide

**DOI:** 10.1192/bjb.2023.15

**Published:** 2024-02

**Authors:** Qamar Hussain, Helen Killaspy, Peter McPherson, Rachel Gibbons

**Affiliations:** 1 University College London, UK; 2 Camden and Islington NHS Foundation Trust, London, UK; 3 Royal College of Psychiatrists, London, UK

**Keywords:** Homicide, clinicians, well-being, support, employer

## Abstract

**Aims and method:**

To investigate the experiences and support needs of consultant psychiatrists following a patient-perpetrated homicide, an anonymous online survey was sent to all consultant psychiatrists registered as members of the UK's Royal College of Psychiatrists.

**Results:**

Of the 497 psychiatrists who responded, 165 (33%) had experienced a homicide by a patient under their consultant care. Most respondents reported negative impacts on their clinical work (83%), mental and/or physical health (78%) or personal relationships (59%), and for some (9–12%) these were severe and long lasting. Formal processes such as serious incident inquiries were commonly experienced as distressing. Support was mainly provided by friends, family and colleagues rather than the employing organisation.

**Clinical implications:**

Mental health service providers need to provide support and guidance to psychiatrists following a patient-perpetrated homicide to help them manage the personal and professional impact. Further research into the needs of other mental health professionals is needed.

Homicide is a rare event. In the year ending March 2021, there were 594 victims of homicide in England and Wales, a rate of around 1/100 000 population.^1^ Most homicides are not committed by people with mental health problems; over the 10 years between 2007 and 2017, 732 (11%) of those convicted of homicide in the UK had a pre-existing mental health diagnosis.^2^ However, although the homicide rate in the general population has fallen since 2005, the proportion of homicides committed by people with a diagnosis of schizophrenia has risen^3^ and many psychiatrists will experience a homicide by a patient at least once in their professional career.^4^ The impact of such an event extends beyond the loss of human life, with far-reaching consequences for the perpetrator, their family and friends and those of the victim.^5–8^ There has been little attention given to the impact on the treating clinicians. One exception is a survey of UK forensic psychiatrists conducted by Mezey and colleagues.^9^ Of the 86 respondents, 26 had experienced at least one patient-perpetrated homicide, with such events evoking intense emotional reactions, including guilt, distress and feelings of subsequent professional and personal isolation. The formal processes that followed were often experienced as promoting a ‘blame culture’, leaving clinicians feeling unfairly judged by systems that have been previously described as being insensitive to the complexities of mental illness and mental healthcare.^10^ We aimed to add to the limited literature in this field by investigating the experiences and support needs of consultant psychiatrists from all disciplines following a homicide by a patient under their care.

## Method

### Survey development

An anonymous online survey was developed using University College London's (UCL) secure ‘Opinio’ platform. The content was adapted from that used in Mezey et al's survey.^9^ Item content and survey structure were reviewed by members of the study team, the UK Royal College of Psychiatrists’ (RCPsych) Working Group on the Impact of Patient Suicide and Homicide on Clinicians, and the RCPsych Forensic Faculty's Executive Committee. The final version comprised 40 descriptive, rateable and free-text items. An initial filter question identified respondents who had experienced a homicide by a patient who was under their care while working as a consultant psychiatrist. If a respondent had experienced more than one such incident, they were asked to focus on the one that had affected them most. The survey covered professional and personal impacts, and the type and sources of support that were offered to help them in the aftermath of the incident, including formal processes (such as internal and external serious incident inquiries, preparing for criminal or coroners’ court hearings) and how helpful any such supports were. Items were worded neutrally to avoid leading respondents. Rateable items assessed respondents’ views using Likert-type scales from 1 to 10 (where higher numbers represented greater impact of the homicide or greater satisfaction with the support received). The survey took approximately 15 min to complete. Anonymity was ensured as no data that could potentially identify respondents were collected and the researchers had no access to respondents’ email addresses.

### Participants

The link to the survey was disseminated by the RCPsych to all consultant psychiatrist members by email on 29 June 2021. The College's most recent workforce survey of 2019 identified around 6000 full-time or part-time members in substantive consultant posts. The survey was available for 1 month, and two reminders were sent, 2 and 3 weeks after the initial email.

### Ethical approval

Approval for the study was obtained from UCL's Research Ethics Committee (reference: 20201/001). Information about the study was provided to participants through the online platform and respondents confirmed their consent prior to completing the survey.

### Data analysis

Survey data were downloaded from the online platform. Simple descriptive statistics were used to collate the quantitative data using SPSS (version 25). Free-text responses were grouped into themes by Q.H. and refined through discussion with the co-authors (H.K., P.M. and R.G.).

## Results

### Response and respondents

A total of 497 psychiatrists responded. Of these, 198 (40%) reported that they had experienced a homicide by a patient under their care, most of whom (165/198, 83%) were consultants at the time and therefore eligible to complete the survey. Their characteristics are summarised in Table 1. Over half (52%) were male. Just under half (49%) were working in general adult psychiatry at the time of the incident; 15% were working in forensic services and 4% in addictions services. The mean time respondents had been working as a consultant was 21 years (s.d. = 9) and most (74%) had been a consultant for over 5 years at the time of the homicide. Over half (51%) reported that the incident had occurred more than 5 years ago and most (60%) reported experiencing only one homicide.

**Table 1:** Characteristics of respondents (*n* = 165) who had experienced a homicide by a patient

Characteristic	*n* (%)
Gender	
Male	85 (52)
Female	40 (24)
Prefer not to say	2 (1)
Missing	38 (23)
Specialty	
Addictions	6 (4)
Child and adolescent	2 (1)
Forensic	24 (15)
General adult	81 (49)
Liaison psychiatry	3 (2)
Mental healthcare of older people	2 (1)
Neuropsychiatry	1 (1)
Psychotherapy	2 (1)
Rehabilitation psychiatry	4 (2)
Prefer not to answer	1 (1)
Other	3 (1)
Missing	36 (22)
Years working as a consultant at time of homicide	
<2	2 (1)
3–5	2 (1)
>5	121 (73)
Missing	40 (24)
Number of homicides experienced	
1	99 (60)
>1	27 (16)
Missing	39 (24)

### Perpetrators and victims

Data on the characteristics of perpetrators were provided by 122 respondents. Most (103/122, 84%) were male. The vast majority (113/122, 93%) of the homicides occurred while the patient was being treated in the community. In 90% (110/122) of cases the incident involved only one victim; two victims were involved in 9/122 (7%) cases and three victims in 3/122 (2%) cases. In most cases (58%), the victim was well-known to the perpetrator, either as a family member (32/122, 26%), friend (27/122, 22%) or partner (18/122, 15%). In 31/122 (25%) of cases the victim was a stranger and in the remaining 24/122 (20%) respondents chose the ‘other’ category (examples included mental health or supported accommodation staff (4), fellow in-patient (2), co-resident in supported accommodation (2), drug dealer (2)).

### Impact of the homicide on the clinician's work

**Fig. 1:**
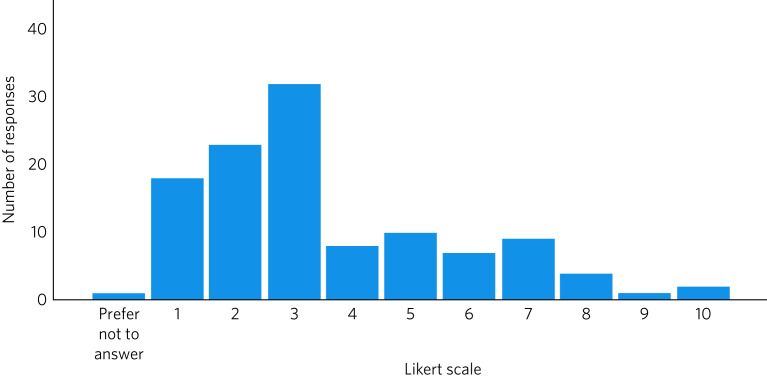
Impact of the homicide on the clinical practice of psychiatrists, measured on a Likert scale of 1 (no effect) to 10 (inability to function effectively).

As shown in Fig. 1, most respondents (95/115, 83%) reported that the homicide had an impact on their clinical practice, with a fifth of those who responded to the question (24/115, 21%) scoring this at least 5 on the Likert scale (mean 3.58, s.d. = 2.2), suggesting a substantial impact.

Of those who gave free-text responses about the type of impact, around half (49/106, 46%) reported a loss of confidence in their professional skills and ability to accurately assess risk, leading to anxiety about making clinical decisions:

‘It caused me to question everything about myself and the structures and processes we have in adult psychiatry – frameworks of care, risk management, roles and responsibilities etc.’

Over half (59, 57%) of the 103 respondents who answered the item on the duration of the impact of the incident on their work stated that it lasted less than 2 years; 35 (34%) reported it as lasting over 2 years and 9 (9%) said that it had a longer-term effect on them.

A few respondents (7/103, 7%) reported that the homicide had a negative impact on their relationships with other members of staff. Some felt colleagues tried to avoid any involvement or blame for the incident:

‘It made me feel unable to trust colleagues who tried to shift blame from themselves.’

### Impact of the homicide on the clinician's mental/physical health

**Fig. 2:**
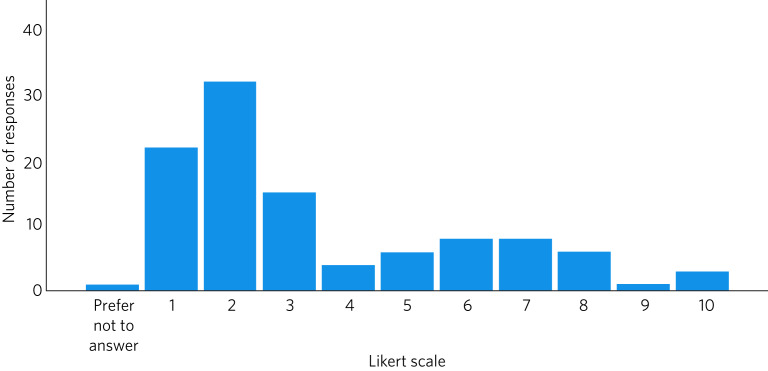
Impact of the homicide on the mental and/or physical health of psychiatrists, measured on a Likert scale of 1 (no effect) to 10 (inability to function effectively).

Figure 2 shows the ratings provided by respondents on the impact of the homicide on their mental and/or physical health. A negative impact was reported by 78% (83/106), with 30% (32/106) rating this at least 5 on the Likert scale (mean 3.5, s.d. = 2.51).

Of the 59 respondents who added free-text comments about the type of mental/physical impact of the homicide, the most common problems were stress, anxiety, depression and sleep disturbance, which were reported by two-thirds (39/59, 66%). The free-text responses provided further insights into the negative psychological impacts that some had experienced:

‘It felt like a personal bereavement but more complicated. I “coped” well in my own view at the time but in fact it was appalling beyond words, […] it has been life-changing for me and contributed to an eventual decision to go part-time and then take early retirement.’

‘I was devastated by the incident and the subsequent enquiry process […] I considered leaving medicine entirely.’

Most respondents (94/106, 89%) reported on the duration of the mental/physical impact; 61/94 (65%) reported it as lasting less than 2 years, 22/94 (23%) as more than 2 years and 11/94 (12%) reported a longer-term impact:

‘The consequences of this homicide lasted throughout my working life. I have felt more easily stressed by adverse working conditions and have found carrying the responsibility for risky patients harder as a consequence.’

### Impact of the homicide on the clinician's personal life

**Fig. 3:**
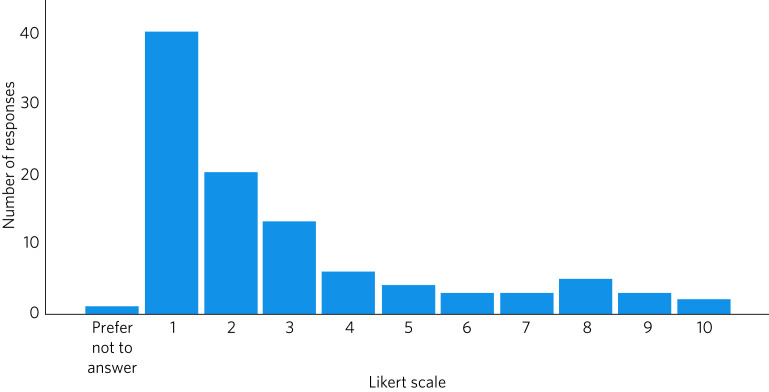
Impact of the homicide on the personal life of psychiatrists, measured on a Likert scale of 1 (no effect) to 10 (inability to function effectively).

Figure 3 shows the results for the 100 respondents who rated the impact of the homicide on their personal life. Over half (59, 59%) reported some impact and a quarter (25, 25%) rated this at least 5 on the Likert scale, but overall, ratings were lower than those assessing the impact on clinical work or mental/physical health (mean 2.92, s.d. = 2.49).

Some respondents noted that holding clear boundaries between their work and home life had mitigated the negative impact:

‘I try hard not to let work issues creep into my personal life.’

However, for an important minority (16, 16%), it was clear that the incident had been highly detrimental to their personal relationships:

‘Divorce. Other relationships broke down. Failure to achieve full professional potential.’

‘It changed who I am. I am scarred by the experience and it caused me to distance myself from my husband to protect him from the details of events. This has never been healed.’

Of the 82 respondents who reported on the duration of the impact of the incident on their personal life, 60 (73%) stated that it lasted less than 2 years, 14 (17%) more than 2 years and 8 (10%) reported it had a longer-term impact.

### Time off work

Most respondents (121/128, 94%) did not take any time off work because of the incident. Some felt they needed to remain at work owing to their heavy workload:

‘Workload is very high in a stretched team, therefore taking time off has impact on patient care, colleague wellbeing and usually results in increased stress on return as nobody else picks up missed clinics, they just get condensed into extended future clinics.’

Others were anxious about being blamed unfairly in their absence:

‘Everyone was trying to cover their back. I needed to be there to stop them pinning it on me.’

However, some reflected that having time off would have been beneficial:

‘In retrospect I ought to have given more weight to the impact of this on me and taken time away. This wasn't suggested though.’

One respondent reported that they later resigned, one retired and three changed specialty as a consequence of the incident.

### Experiences of formal processes

The vast majority of respondents (101/121, 84%) were involved in an internal inquiry into the homicide within their employing organisation and 2% (4/165) were involved in disciplinary proceedings. None of the respondents were referred to the General Medical Council (GMC) or suspended. Most (98/104, 94%) said that the incident was reported in the local media and often in the national press (52/104, 50%), with 14 (13%) being named in media articles and 57 (55%) in an internal or external inquiry report. One-third (32/104, 31%) provided a report for the court about the case and 19 (18%) gave evidence in court.

Some respondents (23/165, 14%) felt that the formal processes were psychologically damaging to them, most commonly feeling that they were unfairly blamed:

‘I assumed the intention was to understand what had happened and not to find a fall guy.’

However, a minority (9/165, 5%) found the formal processes constructive, gaining valuable experience that they could use in the future for themselves or to advise colleagues going through a similar situation. Some also reported that the formal processes validated the decisions they had made in relation to the case:

‘It confirmed my existing beliefs about the importance of accurate record keeping, including formalising leave cover. It was the hardest thing I have ever dealt with but taught me a lot early on. I have been able to support colleagues who have experienced similar things.’

### Clinicians’ experiences of the support offered

Only 60 respondents answered the item about the support offered after the homicide. The majority (40/60, 67%) reported receiving no support from their employing organisation and 28 said that their only support had come from family and friends. The majority of those who were offered support by their employer found this helpful:

‘Very good. Interviewed for 3 h […] by trust lawyer the next day, who drafted my statement that I needed for the next 2+ years […] really wise and helpful.’

‘My team, manager, clinical director and CEO [chief executive officer] were utterly amazing. CEO called me to check in. Team looked after me. Manager called ahead to a meeting I was chairing to make sure they looked after me.’

### Clinicians’ views on the support that should be offered

Table 2 shows the ratings of 100 respondents about the potential helpfulness of different types of support that might be offered. Being contacted by a consultant with expertise in managing such incidents was the most popular suggestion, followed by receiving information about the formal processes that may follow. Most respondents (*n* = 76, 76%) also felt that the RCPsych should have an active role in supporting psychiatrists following such events. Suggestions included: a dedicated webpage with information about what to expect; a support group; signposting to resources to access further help; and publishing guidelines for employing organisations about the support they should provide.

**Table 2:** Respondents’ (*n* = 100) ratings of the potential helpfulness of different support strategies following a homicide by a patient

Support strategy	Not applicable *n* (%)	1–2 (not helpful) *n* (%)	3–4 *n* (%)	5–6 *n* (%)	7–8 *n* (%)	9–10 (extremely helpful) *n* (%)
Being contacted by a consultant colleague with expertise in managing the impact of a homicide by a patient	2 (2)	2 (2)	5 (6)	17 (17)	31 (31)	42 (42)
Receiving information about the formal (internal and external) processes that may follow	3 (3)	1 (1)	6 (6)	20 (20)	33 (33)	37 (37)
Having access to a confidential consultant peer support/reflective group	4 (4)	7 (7)	6 (6)	20 (20)	27 (27)	36 (36)
Having contact from someone who has been through a similar experience to provide guidance and support (e.g. a ‘buddy’ scheme)	4 (4)	8 (8)	12 (12)	17 (17)	28 (28)	31 (31)
Having prior training about the impact and processes following a homicide by a patient	3 (3)	1 (1)	15 (15)	27 (27)	27 (27)	27 (27)
Access to counselling/therapy	6 (6)	24 (24)	22 (22)	21 (21)	16 (16)	11 (11)

### Homicide prediction/prevention and the role of the psychiatrist

Most respondents who answered this question (72/94, 77%) did not feel that the responsibility for predicting and preventing a homicide lay with the consultant psychiatrist. Free-text responses provided explanations for this, including the lack of predictive validity of risk assessments and factors outside the psychiatrist's control, such as resource limitations and pressure to discharge patients from in-patient services to the community:

‘I don't think the Consultant should be seen as responsible. (1) Current risk assessment processes are not accurate enough to predict such rare outcomes, (2) even where there is concern, interventions are not sufficient to reduce risk to zero.’

Some respondents noted the discrepancy between their views on this and those of the public:

‘I think the expectation of others far exceeds our own predictive capabilities.’

## Discussion

This study explored the experiences and support needs of consultant psychiatrists following a patient-perpetrated homicide. We believe that the response to the survey was one of the highest achieved in any RCPsych membership survey and that this is the largest survey of psychiatrists on this topic to date. Nevertheless, the results may be subject to response bias in that those who had particularly negative experiences may have been more likely to participate. Conversely, people may have been less likely to respond if they remained traumatised by the incident. Bearing these limitations in mind, we found that almost half the respondents were general adult psychiatrists and almost all the incidents of homicide occurred while the patient was in the community. Respondents commonly reported negative impacts professionally and personally, including a loss of confidence in making clinical decisions, symptoms of psychological stress and strained personal relationships. Although for the majority, such experiences tended to be relatively mild and short-lived, 20–30% reported they were significantly affected and 9–12% reported long-term negative consequences. Owing to the anonymous nature of our survey, we were unable to explore potential associations between respondents’ ratings of the impact of the incident and individual characteristics, but plausible explanations for the variation include psychological resilience, the relationship with the patient,^11^ the response of the employing organisation, media interest and the nature of the homicide itself. Our results are consistent with Mezey et al's survey of forensic psychiatrists^9^ and a study of the impact of patient suicide on Flemish and Swiss mental health professionals.^12,13^

Although the self-report scale we used to assess the impact of the homicide was not scored in a way that captured positive impacts, in contrast to previous studies some of our respondents’ free-text responses illustrated that they felt they had gained valuable experience from the incident that could be used to support colleagues experiencing something similar.

Few took time off work, which is of concern given that Howard et al's systematic review of personal resilience among psychiatrists^11^ reported higher levels of burnout and psychological distress than in other physician groups and identified serious incidents such as patient suicide to be particularly toxic for emotional health.

Similar to Mezey et al's findings,^9^ the formal processes following the homicide were usually experienced as distressing, unfairly blaming of individuals and rarely constructive. Given that none of our respondents were referred to the GMC, the system does appear unjust. Holliday et al,^14^ in their review of formal inquiries following patient-perpetrated homicides in New Zealand, concluded that they often resulted in individuals or teams being judged harshly in what they referred to as ‘hindsight bias’. Similarly, Alexander et al^15^ recommended that formal processes following a serious incident should be ‘conducted in a constructive climate which is geared towards learning rather than a blame culture which may cultivate additional stress’. Moreover, it has been suggested that since homicides are extremely complex events, it may be more beneficial to reflect on the incident openly with a view to learning, rather than assuming that this process can prevent future tragedies.^8,16^

Our findings concur with previous studies in showing that many clinicians rely on family or friends for support after a serious incident.^17,18^ However, our respondents were also keen to be offered more formal support and information to help them deal with the emotional impact and guide them through the subsequent formal processes. Similar to findings from a survey of psychiatrists’ experiences following a patient suicide,^19^ popular suggestions included having access to a colleague with expertise in managing the impact, a fellow consultant who could ‘buddy’ them through the aftermath or a peer support group.

### Implications

This survey provides evidence of the mainly negative professional and personal impact of patient-perpetrated homicide on psychiatrists. It clearly demonstrates the need to develop guidance on the support that should be offered by employing organisations and the RCPsych. Further research is needed to investigate the impact and support required for other mental health professionals.

## About the authors

**Qamar Hussain** is an MSc student in clinical mental health studies in the Division of Psychiatry, University College London, UK. **Helen Killaspy** is Professor of Rehabilitation Psychiatry in the Division of Psychiatry, University College London, UK. **Peter McPherson** is a PhD student in the Division of Psychiatry, University College London, UK. **Rachel Gibbons** is Chair of the Working Group on the Impact of Patient Suicide and Homicide on Clinicians, Royal College of Psychiatrists, London, UK.

## Data Availability

Data are available from the corresponding author, H.K., on reasonable request.

## References

[1] Office for National Statistics. Homicide in England and Wales: Year Ending March 2021. ONS, 2022 (https://www.ons.gov.uk/peoplepopulationandcommunity/crimeandjustice/articles/homicideinenglandandwales/yearendingmarch2021).

[2] The National Confidential Inquiry into Suicide and Homicide by People with Mental Illness. Annual Report: 2019. England, Northern Ireland, Scotland, and Wales. University of Manchester, 2021 (https://sites.manchester.ac.uk/ncish/reports/annual-report-2021-england-northern-ireland-scotland-and-wales/).

[3] Flynn S, Ibrahim S, Kapur N, Appleby L, Shaw J. Mental disorder in people convicted of homicide: long-term national trends in rates and court outcome. Br J Psychiatry 2021; 218: 210–6. 32624025 10.1192/bjp.2020.94

[4] Botelho M, Gonçalves RA. Why do people kill? A critical review of the literature on factors associated with homicide. Aggress Violent Behav 2016; 26: 9–15.

[5] Ng L, Merry AF, Paterson R, Merry SN. Families of victims of homicide: qualitative study of their experiences with mental health inquiries. BJPsych Open 2020; 6(5): e100. 10.1192/bjo.2020.84 PMC7488330 32873366

[6] Ng L, Merry AF, Paterson R, Merry SN. Clinicians’ experiences of inquiries following mental health related homicide: a qualitative study. Australas Psychiatry 2022; 30: 185–9. 33939929 10.1177/10398562211009260 PMC8988458

[7] van der Ploeg E, Dorresteijn SM, Kleber RJ. Critical incidents and chronic stressors at work: their impact on forensic doctors. J Occup Health Psychol 2003; 8: 157–66. 12703881 10.1037/1076-8998.8.2.157

[8] Szmukler G. Homicide inquiries: what sense do they make? Psychiatr Bull 2000; 24: 6–10.

[9] Mezey G, Rowe R, Adshead G. Impact of homicide by a psychiatric patient on forensic psychiatrists: national survey. Psychiatr Bull 2021; 45: 183–9. 10.1192/bjb.2020.96 PMC9059313 35346405

[10] Clarke I. Learning from critical incidents. Adv Psychiatr Treat 2008; 14: 460–8.

[11] Howard R, Kirkley C, Baylis N. Personal resilience in psychiatrists: systematic review. BJPsych Bull 2019; 11: 209–15. 10.1192/bjb.2019.12 PMC12402926 30855001

[12] Rothes IA, Scheerder G, Van Audenhove C, Henriques MR. Patient suicide: the experience of Flemish psychiatrists. Suicide Life Threat Behav 2013; 43: 379–94. 23530711 10.1111/sltb.12024

[13] Finlayson M, Graetz Simmonds J. Impact of client suicide on psychologists in Australia. Aust Psychol 2018; 53: 23–32.

[14] Holliday E, Taylor PJ. Consequences for clinicians and mental health services of a homicide by a current or recent patient: a European Union (EU) wide survey. Int J Forensic Ment Health 2015; 14: 218–29.

[15] Alexander DA, Klein S, Gray NM, Dewar IG, Eagles JM. Suicide by patients: questionnaire study of its effect on consultant psychiatrists. BMJ 2000; 320: 1571–4. 10845964 10.1136/bmj.320.7249.1571 PMC27400

[16] Petch E, Bradley C. Learning the lessons from homicide inquiries: adding insult to injury? J Forensic Psychiatry 1997; 8: 161–84.

[17] Lee J, Ogloff JR, Daffern M, Martin T. The impact of inpatient homicide on forensic mental health nurses’ distress and posttraumatic stress. Int J Forensic Ment Health 2015; 14: 93–100.

[18] Malik S, Gunn S, Robertson N. The impact of patient suicide on doctors and nurses: a critical interpretive meta-synthesis. Arch Suicide Res 2022; 26: 1266–85. 33631083 10.1080/13811118.2021.1885533

[19] Gibbons R, Brand F, Carbonnier A, Croft A, Lascelles K, Wolfart G, et al. Effects of patient suicide on psychiatrists: survey of experiences and support required. BJPsych Bull 2019; 43: 236–41.

